# Hydrogel membranes based on genipin-cross-linked chitosan blends for corneal epithelium tissue engineering

**DOI:** 10.1007/s10856-012-4666-7

**Published:** 2012-05-09

**Authors:** Maria Grolik, Krzysztof Szczubiałka, Bogumił Wowra, Dariusz Dobrowolski, Bogusława Orzechowska-Wylęgała, Edward Wylęgała, Maria Nowakowska

**Affiliations:** 1 Faculty of Chemistry, Jagiellonian University, Ingardena 3, 30-060 Kraków, Poland; 2 Department of Ophthalmology, District Railway Hospital, Panewnicka 65, 40-760 Katowice, Poland; 3 Department of Maxillo-Facial Surgery, Silesian Medical University, Filarowa 5A, 40-565 Katowice, Poland

## Abstract

Novel polymeric hydrogel scaffolds for corneal epithelium cell culturing based on blends of chitosan with some other biopolymers such as hydroxypropylcellulose, collagen and elastin crosslinked with genipin, a natural substance, were prepared. Physicochemical and biomechanical properties of these materials were determined. The in vitro cell culture experiments with corneal epithelium cells have indicated that a membrane prepared from chitosan–collagen blend (Ch–Col) provided the regular stratified growth of the epithelium cells, good surface covering and increased number of the cell layers. Ch–Col membranes are therefore the most promising material among those studied. The performance of Ch–Col membranes is comparable with that of the amniotic membrane which is currently recommended for clinical applications.

## Introduction

Ocular diseases and wounds requiring treatment affect more than 15 million people worldwide each year [1]. A substantial fraction of them are mechanical, thermal, or chemical injuries of cornea. It is estimated that more than 10 million people in the world suffer from problems with cornea [2] which are currently the second most common cause of blindness in the world, with only cataract being more frequent. Cornea is the outermost transparent five-layer part of the eyeball covering iris and pupil. It plays three main important roles. First, it acts as a physical barrier against pathogenic microorganisms, dirt, and other noxious physical factors. Second, it plays an active role in the process of vision by refracting light onto lens and retina. It is estimated that cornea is responsive for 70 % of the refracting power of an eye [3]. Third, it absorbs UV radiation between 200 and 295 nm preventing the damage of other elements of the optical system of an eye. Corneal transparency and optical refraction is preserved as a consequence of the continuous renewal of the epithelium, the outermost layer of the cornea [4]. Epithelium is made up of 5–7 layers of very regularly arranged cells [5]. The thickness of human corneal epithelium is about 50–52 μm while overall thickness of the cornea is about 600 μm. The renewal of corneal epithelium is maintained by the proliferation and differentiation of the corneal epithelial stem cells, or limbal stem cells (LSCs) located in the basal layer of the cornea, known as the limbus, located at the border of cornea and sclera [6, 7]. Cornea is quite resistant to minor injuries or abrasions due to the ability of the corneal epithelium to undergo continuous renewal. In the case of injury, the epithelial cells migrate at a rate of 60–80 μm/h until wound is closed [8]. Dysfunction or loss of the LSCs resulting from chemical or thermal burns, contact lenses related or microbial infections, inflammatory eye diseases, hereditary or iatrogenic disorders can cause the cornea surface opaqueness [6, 7, 9].

There are several approaches to the treatment of seriously injured cornea. One of them is a replacement of the cornea. Corneal blindness may be treated by transplantation of donor cadaver corneas, known as penetrating keratoplasty [3]. In fact, it is cornea which was the first allografted human tissue [10] and penetrating keratoplasty is still one of the most successful types of transplantations. However, the availability of donor corneas is very limited. Moreover, in some cases such as severe chemical burns, ocular pemphigoid, Stevens–Johnson syndrome, trachoma, severe dry eye syndrome, severe herpes zoster, aniridia, certain metabolic opacities, ectodermal dysplasia, and vascularized traumatic injuries, penetrating keratoplasty gives poor results [3].

Damage of the surface epithelia and corneal stroma leads to the severe cicatrisation of the ocular surface. In such cases combination of artificial materials (PMMA) and human solid tissue is used to restore vision. Currently, in severely destroyed corneas application of keratoprosthesis is recommended [11]. That device, applicable in clinical practice, is built from optical cylinder and its carrier. Currently, two types of keratoprostheses are used. The most popular are the Boston type 1 and 2 keratoprostheses carried by the donor’s cornea [12], while the second type, called osteo-odonto-keratoprosthesis [13], is carried by the skeletal bone or dental laminate. Also the artificial corneas, e.g., Alphacor made from PHEMA are available. They have a sponge-like peripheral region with interconnecting pores allowing biointegration with surrounding corneal tissue [14]. Corneal replacements made of animal tissues, usually porcine, are also used [15, 16].

Corneal structure reconstruction can be also proceeded in a layer by layer approach. Transparent corneal surface may be restored by transplantation of autologous limbal or oral mucosa epithelia cultured ex vivo on a proper support which is then implanted together with the confluent sheet of expanded epithelial cells. This procedure is sufficient to reconstruct the ocular surface, however, for the reconstruction of deeper corneal layers penetrating or lamellar keratoplasty techniques are required. Various materials have been used as LSCs culture supports, the amniotic membrane (AM) being the clinical standard due to the content of growth factor and low immunogenicity [9]. However, this material is costly and is associated with a high risk of disease transmission. Therefore, alternative materials for AM are strongly desired. Both synthetic and natural polymers are considered as AM replacements for the ex vivo culturing of corneal cells. The examples of the former are modified and unmodified copolymers of 2-hydroxyethyl methacrylate (HEMA) and methyl methacrylate (MMA) [2]. Natural polymers used for the fabrication of scaffolds include gelatin and chondroitin sulfate [17], silk fibroin [18], recombinant human collagen [19], and argon plasma treated collagen [20].

The biopolymer, which has gained great and still rapidly rising interest in ophthalmology, is chitosan (Ch). Chitosan is a linear polysaccharide derived by partial N-deacetylation of chitin, which is the primary structural polymer in arthropod exoskeletons, shells of crustaceans, or the cuticles of insects [21]. It is extensively studied due to its unique biocompatibility, biodegradability, biological inertness, stability in the natural environment as well as antifungal and anti-bacterial properties [22, 23]. It has found numerous pharmaceutical applications, primarily as a component of drug delivery systems including ocular ones [24–31]. We have also studied the application of chitosan-based materials as a drug-carrier [32] and as antiheparin agents [33, 34]. What is important from the point of view of the studies presented here, chitosan is successfully used for constructing supports for adhesion, proliferation, and differentiation of cells [35–37].

To increase their mechanical strength cell culture supports based on chitosan are chemically crosslinked usually using glutaraldehyde [38, 39], but also with reagents such as glyoxal [40] and epichlorohydrin [41]. However, these substances are toxic and may impair the biocompatibility of the crosslinked biomaterials. Therefore, much interest is now directed toward natural crosslinking substances with low toxicity such as genipin (Gp), which was used to crosslink all the materials described in this paper. Genipin is naturally found in the *Gardenia jasminodes Ellis* fruit. Genipin-crosslinked chitosan is a fluorescent bluish hydrogel, which has been intensively studied recently [29, 42–45] since it is reported to be about 5,000–10,000 times less cytotoxic than glutaraldehyde [46] and genipin-cross-linked materials have comparable mechanical strength to the glutaraldehyde-cross-linked ones [47].

The purpose of the current studies was to obtain genipin-crosslinked chitosan-based scaffolds and to determine their applicability as alternatives for AM in reepithelialization of the cornea. Although chitosan and its derivatives, both as a single polymer and in blends with other polymers, have been already used as supports for corneal epithelial cells, they were not chemically crosslinked [14, 48], or crosslinked with toxic [49] or costly [50] crosslinkers. To the best of our knowledge, this is the first report on the application of genipin-crosslinked chitosan scaffolds for culturing corneal epithelium. We have studied the chitosan supports containing additions of other biopolymers, i.e. hydroxypropyl cellulose (HPC), collagen (Col), and elastin (Ela) frequently used for the fabrication of scaffolds.

## Experimental section

### Materials

Low-molecular-weight chitosan (Ch) was purchased from Sigma. The degree of deacetylation of the chitosan was approximately 77 %, as determined by elemental analysis. Genipin (Gp) powder (98 %) was obtained from Challenge Bioproducts Co. Hydroxypropyl cellulose (HPC), elastin (Ela), and boric acid (ACS reagent) were obtained from Sigma. Solution of collagen type I (0.3 %, Col) from rat tail was obtained from BD Biosciences. Disodium hydrogen phosphate (analytical grade) and potassium dihydrogen phosphate (analytical grade), hydrochloric acid, ethanol (analytical grade) were obtained from Polskie Odczynniki Chemiczne (Gliwice, Poland). Sodium tetraborate decahydrate (analytical grade) was obtained from Fluka. Sodium chloride (analytical grade) was obtained from Lach:Ner. All chemicals were used without further purification. Water was distilled twice.

### UV–Vis absorption spectra

The UV–Vis absorption spectra of the membranes supported on 1-mm thick quartz plates were measured using a 8452A Hewlett-Packard spectrophotometer.

### Preparation of membranes based on chitosan

Chitosan (Ch) solution (2 % w/v) was prepared by dissolving 0.8 g of Ch in 40 mL of 0.1 M hydrochloric acid. Genipin (Gp) solution (5 % w/v) was prepared by dissolving 0.1 g of Gp powder in 2 mL of 70 % v/v ethanol. 6 % w/v Hydroxypropylcellulose (HPC) solution was prepared by dissolving 0.9 g of HPC powder in 15 mL of water. Elastin (Ela) solution (13.3 % w/v) was obtained by dissolving 2 g of Ela in 15 mL of 0.25 M oxalic acid. Collagen solution was used as received. The hydrogel membranes were prepared using 1.5 mL of clear, slightly yellowish mixture of equal volumes of Ch solution and the solutions of HPC, Col and Ela, respectively. The polymeric mixtures were stirred for 5 min and then 40 μL of Gp solution was added to initiate the crosslinking reaction. The mixture was homogenized by vigorous stirring for 10 min at room temperature and then poured onto a 60 mm plastic Petri dish and placed in an incubator for 48 h at 45 °C. After a few of hours the solution became lightly blue and increasingly viscous due to the started crosslinking reaction.

### Swelling ratio measurements

The swelling characteristics of the crosslinked chitosan hydrogels were determined by swelling the membranes at various pH values (6.0, 7.4, and 9.0) at room temperature. The round-shaped membrane 60 mm in diameter was immersed in a Petri dish containing 10 mL of PBS buffer. After soaking for 24 h, the sample was removed, carefully drained with a filter paper to remove excess of liquid, and immediately weighed. In a separate experiment it was determined that swelling process reached equilibrium within 24 h. The swelling ratio of the membrane (*S*) was calculated according to the well-known equation:

1 $$ S = \frac{{W_{\text{s}} - W_{\text{o}} }}{{W_{0} }} \times 100\% $$

where *W*_s_ is the weight of the swollen membrane and *W*_0_ is the weight of the dry membrane. Each swelling measurement was repeated three times and the average values are reported.

### Contact angle measurements

The values of the contact angle of water on polymer membranes were measured using Surftens Universal instrument (OEG GmbH, Frankfurt, Germany) at room temperature. A small drop of doubly distilled water was deposited onto the membrane and the contact angle was measured immediately. The contact angle values reported are the averages of five consecutive measurements for each sample.

### Optical microscopy

The Nikon Eclipse LV 1000 optical microscope was employed to observe the morphologies of the membranes based on Ch crosslinked with Gp. The membranes were imaged at room temperature.

### Atomic force microscopy (AFM)

The surface topography of the membranes was analyzed using a Nanoscope IVA atomic force microscope. AFM images in air were obtained using tapping mode technique. The root mean square (RMS) roughness was calculated from data obtained.

### Mechanical testing

Mechanical measurements of the membranes were carried out on a computerized testing machine Zwick 1435 (Zwick GmbH & Co., Ulm, Germany). The rectangular membrane samples (50 × 5 mm) were analyzed at room temperature in air. The membranes were placed in the sample holder of the machine and stretched at a constant rate of 10 mm/min. The tensile strength of a membrane (*R*_*r*_), i.e. the maximum stress a membrane can withstand while being stretched or pulled before necking, was estimated using the following equation:

2 $$ R_{r} = \frac{{F_{r} }}{A}[{\text{MPa}}] $$

where *F*_*r*_ is the load at a destruction moment [N] and *A* is the cross sectional area of the membrane.

The second parameter determined was the elongation at break. It is the amount of uniaxial strain at fracture. To determine the percent of elongation at break fractured membrane was removed from the grips. Data were fitted to the following equation:

3 $$ A = \frac{\Updelta L}{{L_{0} }} \times 100\% $$

where *A* is the percentage elongation at break of the membrane sample, Δ*L* is the increase of the sample length and *L*_0_ is the length of original membrane sample. Each mechanical measurement was repeated ten times and the average values are reported.

### Cell culture assays

The agreement of the Bioethical Commission of Silesian Medical University was obtained (agreement number: NN-6501-184/I/05/06).

Culture media and chemicals were purchased from Sigma (Germany). Reagents for immunostaining were purchased from Santa Cruz Biotechnology Inc. (USA). All parts of the experiment were performed under tenets of Declaration of Helsinki.

The cells used in the study were human corneal epithelial cells collected for cultivated epithelium transplantation procedure. The limbal epithelium source were the eyes of healthy donors. Before donation each eye was examined to detect pathology which could pose a potential risk of visual acuity decrease in the future. All patients were informed about transplantation procedure, experimental assays, and signed agreement forms.

Limbal epithelium was collected under local anesthesia with local decontamination with 10 % solution of povidone–iodine for skin and 5 % povidone–iodine for conjunctiva. One minute after decontamination agent was washed out with a buffered salt solution (BSS). Limbal 2 mm^2^ specimen from upper limbus was gently cut with a crescent knife. Tissue was transferred to corneal storage medium at 4 °C. Tissue specimen was then trypsinized to obtain cell suspension with 1 % trypsin and 0.01 % EDTA for 10 min. Cells were gently scraped with the microscraper.

Culture dishes (Becton–Dickinson, USA) were covered with 3T3 fibroblasts (ATCC, USA) a week before the test. Cells were cultivated in Dulbecco’s Modified Eagle’s Medium (DMEM) with 10 % bovine serum and penicillin/streptomycin mixture. The monolayer of 3T3 fibroblasts was inactivated by incubation in regular medium containing 2 μg/ml of Mitomicin C for 2 h. The whole epithelial culture was carried out in the presence of 3T3 fibroblasts as a source of growth factors. The epithelial single cells were seeded on the membranes of two types (Ch–Col and Ch–Ela) in Petri dishes of 100 mm diameter. Cellular suspension with density of 1–4 × 10^4^ cells for 1 mL were settled in the culture dishes (Cell counter, Coulter Z1, Miami, USA). Epithelial cultures were carried out in standard conditions in 37 °C in humidified atmosphere of 5 % CO_2_ and 95 % air. The medium was supplemented DMEM/HAM F12 mixture with 10 % bovine serum, 0.5 % dimethyl sulfoxide (DMSO), 10 ng/ml mouse epidermal growth factor (EGF), 5 μg/mL bovine insulin, 0.1 nM cholera toxin, 0.18 mM adenine, 2 nM triiodothyronine, 4 mM l-glutamine, 0.4 mg/mL hydrocortisone, and 100 μg/mL penicillin and streptomycin mixture. Culture medium was changed every 48 h. At the10th day of culture the plates were inspected under the light microscope for evaluation of epithelial growth [51].

The histological examinations of the samples were carried out. For these investigations the membranes with cultured cells were fixed with 10 % neutral buffered formalin (4 % formaldehyde in phosphate buffered saline) overnight at 4 °C. To remove fixative agent and water the samples were dehydrated in a graded series of alcohol solutions (10–20–50–95–100 %). Finally, in order to visualize and differentially identify microscopic structures of cultured epithelium the histological stains (hematoxylin—blue and eosin—pink) were used. Immunostaining for cytokeratin 3 (K3), cytokeratin 12 (K12), protein p63, and connexin 43 was performed to confirm corneal origin of the epithelium (K3, K12) and the presence of low differentiated cells.

## Results and discussion

**Fig. 1:**
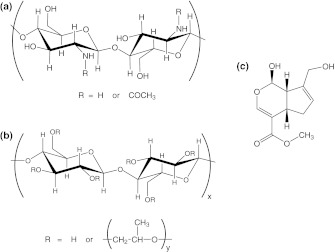
Structures of **a** chitosan, **b** hydroxypropyl cellulose, **c** genipin

The polymers used in this study include two polysaccharides, i.e. chitosan (Ch) and hydroxypropyl cellulose (HPC) (Fig. 1) and two proteins, i.e. collagen (Col) and elastin (Ela).

**Fig. 2:**
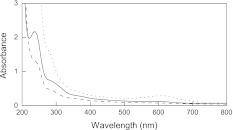
Absorption spectra of Ch–HPC (*solid line*), Ch–Col (*dashed line*), and Ch–Ela (*dotted line*) membranes supported on the quartz plates. The thickness of the membranes was 23 ± 5, 6 ± 2, and 9 ± 3 μm, respectively

Ch contained in the membranes was crosslinked with genipin (Gp). In the case of membranes obtained from Ch and Col, both polymers could be crosslinked with Gp, while there are no reports suggesting the possibility of crosslinking Ela with Gp. Transparent membranes were obtained which are slightly bluish-brown when dry and bluish when hydrated. This is due to their absorption at about 610 nm (Fig. 2).

Since the membranes are expected to be resorbed after implantation, the fact that they are colored upon implantation should not pose a problem. On the contrary, their bluish tint should facilitate visual estimation of the degree of their resorption. The thickness of the membranes obtained was in the range from 6 to 23 μm.

### Swelling of membranes

Degree of swelling is an important parameter of the corneal culture scaffolds. It was also important to determine the swelling equilibration time for the studied materials because the specimens used as cell scaffolds are ultimately implanted into the eye therefore their size should not undergo considerable changes. All membranes revealed a rapid initial weight increase in the PBS buffer and reached an equilibrium within approximately 10 h. The swelling ratios of the Ch–HPC, Ch–Col, and Ch–Ela membranes at different pH values are presented in Table 1.

**Table 1:** Swelling ratios, *S* (%), of the membranes at different pH values determined after 24 h of equilibration

Membrane material	S (%)		
	pH = 6.0	pH = 7.4	pH = 9.0
Ch–HPC	297	142	415
Ch–Col	348	774	441
Ch–Ela	202	620	460

All polymeric hydrogel membranes display significant water sorption ability. The samples prepared from the mixtures of Ch with proteins (Col and Ela) reveal similar degree of swelling. At pH = 7.4 the values of swelling ratio were very different for the three materials studied, while for pH of 6 and 9 the differences were much smaller. The swelling behavior of Ch crosslinked with Gp has been already well characterized [29]. The degree of swelling of genipin-crosslinked Ch was found to increase with decreasing of pH value. That can be explained considering the pH effect on the protonation-deprotonation of the amino groups present in chitosan macromolecule inducing conformational changes of macromolecule in the networks. Protonation of amino groups in acidic solutions leads to the chain extension and chain repulsion. That increases the amount of water present in the polymeric network. HPC present in the chitosan gel (Ch–HPC) decreases the Ch sorption ability and lowers hydrogel sensitivity to the pH of solution. However, the membranes containing Col, Ela, and HPC do not follow the pH dependence of swelling characteristic of Ch.

### Contact angle measurements

It is known that the hydrophilicity/hydrophobicity of a biomaterial is one of its most important parameters which determines the quality of cell adhesion and the rate of their proliferation. It was found that the cell attachment to the surfaces is enhanced when the surfaces are hydrophilic. Enhanced cell attachment is favored by the polar interactions (i.e. hydrogen bonding) between hydrophilic functional groups at the polymer surface and cell membrane proteins. Since the polysaccharides, i.e. Ch and HPC, used for the fabrication of the membranes are both hydrophilic (HPC becomes hydrophobic only above about 42 °C, which is its lower critical solubility temperature (LCST), well above the physiological temperature) while the proteins, i.e. Col and Ela, are hydrophobic, it is difficult to predict the hydrophilicity of the blends composed of a polysaccharide and a protein material. Therefore, water contact angle measurements were performed for the membranes using the sessile drop method. The results are shown in Table 2. The contact angles for all the membranes studied do not differ within the experimental error and range from about 55° to 62°.Thus, the contact angle measurements indicate that all the materials studied are moderately hydrophilic, are compatible with hydrophilic corneal surface, and can be potentially used as corneal epithelium culture supports.

**Table 2:** Water contact angle values for the studied membranes

Membrane material	Contact angle (°)
Ch–HPC	60.28 ± 4.13
Ch–Col	60.40 ± 7.81
Ch–Ela	54.94 ± 5.48

### Surface morphology studied with optical microscopy

**Fig. 3:**
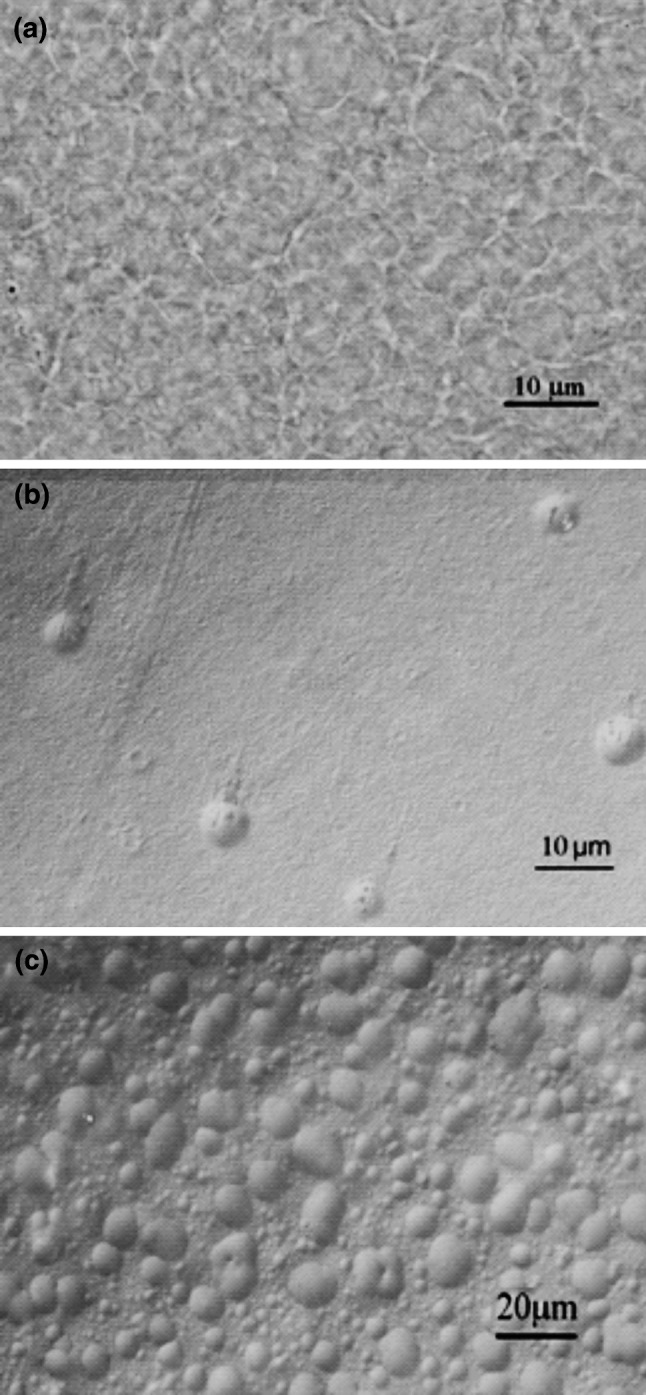
The optical microscopic images of the surface of the dry membranes **a** Ch–HPC, **b** Ch–Col, and **c** Ch–Ela. Magnification: ×50

Except for the surface chemistry, surface morphology is also an important factor in cell attachment mechanism [52, 53]. The surface roughness increases the effective surface area resulting in enhanced interactions between the cells and the polymer surfaces. It was found that the effects of the presence of the pores and the surface hydrophilicity on the migration rate of the corneal epithelial cells were additive [54]. The microscopic examinations of the membranes based on chitosan cross-linked by genipin revealed their very different morphologies (see Fig. 3).

For Ch–HPC (Fig. 3a) the surface seems to have a fibrous structure, while the morphology of Ch–Col surface is very smooth, with only some defects visible (Fig. 3b). The Ch–Ela membranes (Fig. 3c) are covered with droplet-like hemispherical features. Thus, by the addition of another biopolymer to the chitosan one can obtain genipin crosslinked membranes of very different morphologies. This is an important finding indicating that the surface morphology of the chitosan membranes may be easily modified and optimized for corneal epithelium growth and migration.

### Surface morphology studied with AFM

**Fig. 4:**
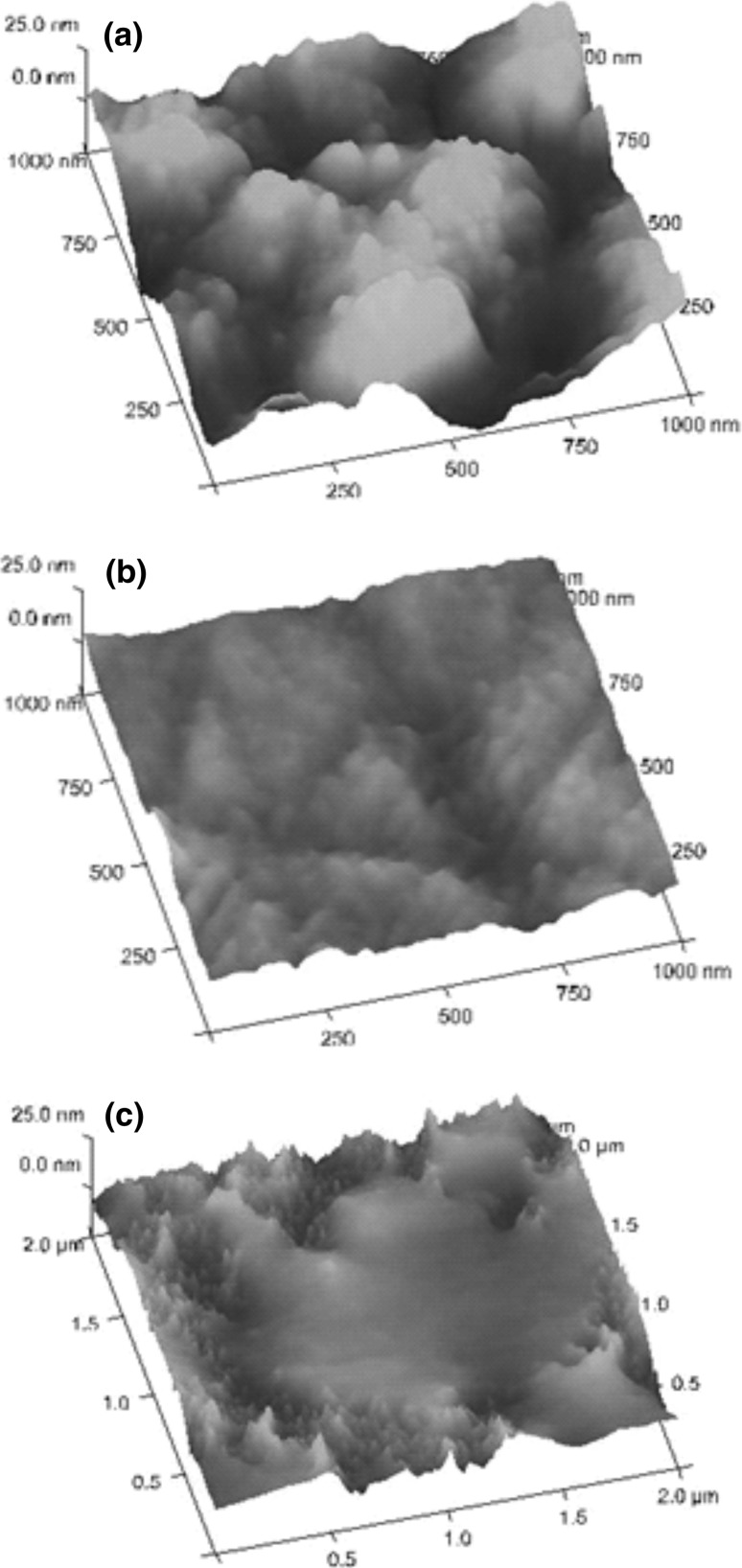
The AFM images of surface of **a** Ch–HPC, **b** Ch–Col, and **c** Ch–Ela membranes

AFM is increasingly often used in the studies of both ocular surface [55] and the surface of corneal epithelium scaffolds [20]. These measurements allow close observation of surface topography and the quantitative determination of surface roughness. The AFM images of the membranes are presented in Fig. 4. It can be seen that the surfaces of Ch–HPC membranes display large objects (Fig. 4a) while the surface of Ch–Col and Ch–ELa membranes is much smoother (Fig. 4b, c). The morphology of Ch–HPC surface is visually very similar to that found for anterior basement membrane of human cornea [51]. The values of RMS roughness of the membranes obtained from the AFM measurements are given in Table 3. The membranes based on Ch with proteins are quite smooth, with much lower RMS roughness than that of Ch–HPC membrane.

**Table 3:** Values of the RMS roughness (nm) of the studied membranes

Membrane material	RMS roughness (nm)
Ch–HPC	9.41
Ch–Col	2.72
Ch–Ela	3.71

### Biomechanical testing

The materials which can be used to construct the membranes applied as supports for corneal epithelium are particularly mechanically demanding. On one hand, they must be strong enough to survive prolonged immersion in the cell culture liquid medium and the implantation procedure, usually by suturing. On the other hand, they are expected to biodegrade after a confluent layer of the epithelial cells, introduced together with the support, covers the cornea. Too low degree of crosslinking results in formation of very fragile membrane while to high degree of crosslinking may render the biodegradation period excessively long. Therefore, the quality of a membrane is a result of a compromise between its mechanical properties and biodegradability. The membranes for epithelial grafts carriers prepared in current studies were mechanically characterized and the results are shown in Table 4.

**Table 4:** Values of tensile strength, elongation at break, and Young’s modulus of the membranes

Membrane material	Tensile strength (MPa)	Elongation at break (%)	Young’s modulus (GPa)
Ch–HPC	31.70 ± 4.16	0.32 ± 0.04	19.93 ± 3.32
Ch–Col	46.93 ± 5.72	0.36 ± 0.05	23.53 ± 4.22
Ch–Ela	48.10 ± 5.76	0.28 ± 0.05	33.03 ± 5.79

The Young’s modulus was determined as a slope of the linear region of the stress–strain diagram at very small elongations

The values of tensile strength for the materials obtained (32–48 MPa) are much higher than those for the scaffolds obtained from amniotic membrane (2.3 MPa) [56] or decellularized porcine cornea (2.4–4.2 MPa) [12]. The elongation at break expresses the elasticity of a material and it is very similar for all samples studied. It was concluded that the blends containing proteins are the most promising candidates as cell culture supports. Therefore, cell culture tests were performed using the Ch–Col and Ch–Ela membranes.

### Epithelial cell culture tests

**Fig. 5:**
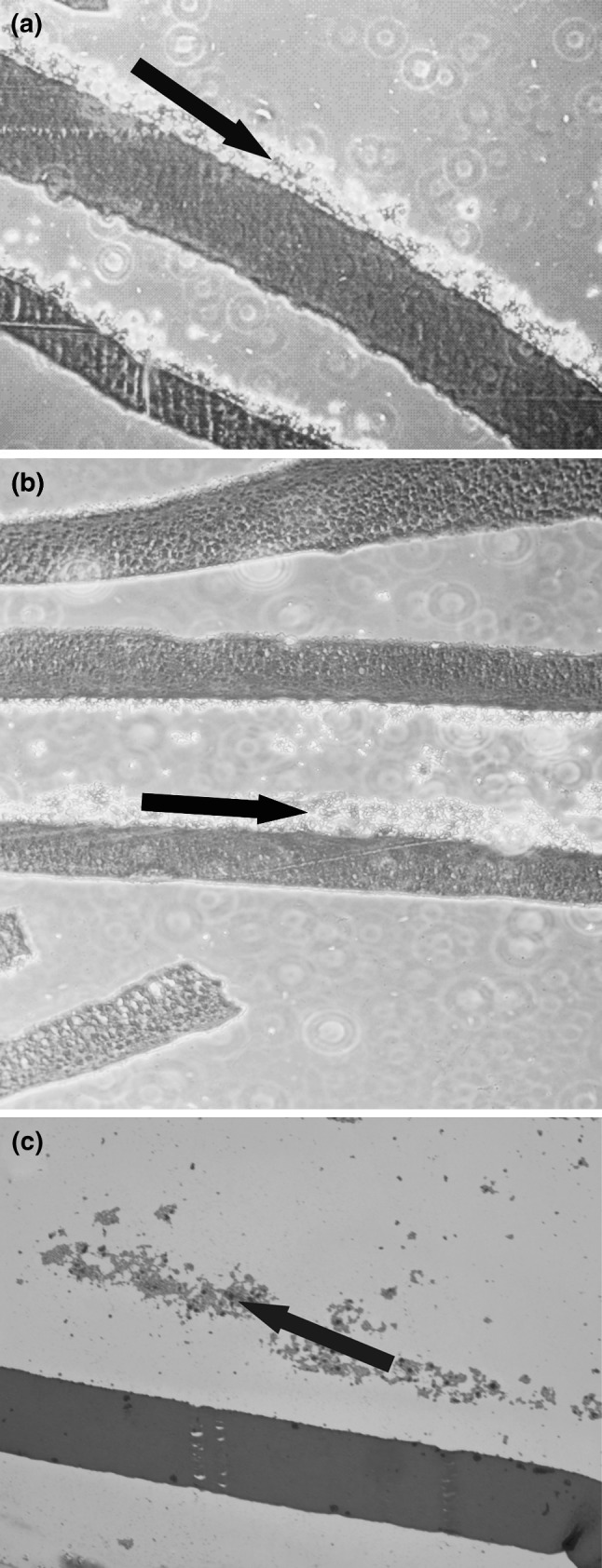
The histologic specimens of **a** Ch–Col, **b** amniotic membrane, and **c** Ch–Ela membranes. Magnification: ×50

In the majority of the cultures carried out on Ch–Col membranes we received regular stratified growth of the cultivated epithelium with good surface covering (Fig. 5a). We observed unusual number of epithelial layers (up to nine, compared with five layers typical of human epithelium) which could be ascribed to the culture conditions. In histologic specimens it was shown that overgrown layers are poorly adherent if compared with basally located cells. Results were comparable with standard cultures carried out on the amniotic membrane (Fig. 5b) dedicated for clinical application [57]. Koizumi et al. [58] reported that denuded amniotic membrane allows also to receive multilayer epithelia with at least five layers of cells.

In the case of Ch–Ela membranes assay (Fig. 5c), growth was not regular with differences in the number of cell layers, poor attachment to the carrier surface and local areas covered only by epithelial colonies. Therefore, only Ch–Col carriers can be considered as eligible for grafting in humans. Membrane compounds require further studies to establish proper surface structure able to carry stratified epithelium. Collagen, which is a common component of basement membranes, seems to be more efficient in improving adhesive properties of Ch–Col membranes. The design of the artificial membranes should include superficial features of human basement membranes to obtain adequate and long-lasting cellular attachment.

## Conclusions

Novel polymeric membranes based on blends of biopolymers Ch–HPC, Ch–Col and Ch–Ela crosslinked with natural substance, genipin, have been successfully prepared with the aim to use them as supports for corneal epithelium cell culturing. Due to the poor biomechanical performance of Ch–HCP that material was eliminated from the biological studies. The cell culture experiments carried out on Ch–Col and Ch–Ela membranes have indicated that Ch–Col is the most promising material. The results obtained with Ch–Col were comparable with these of standard cultures carried on the amniotic membrane, currently recommended for clinical applications. The good performance of Ch–Col can be explained considering the chemical properties of the biopolymers used but also good physicochemical and biomechanical characteristic of Ch–Col membrane, especially reasonable hydrophilicity, optimal morphology and reasonable mechanical parameters, all most likely resulted from good mixing of the blend components forming the homogenous mixture and the fact that both components undergo crosslinking process. Thus, genipin crosslinked Ch–Col hydrogel seems to be a promising material for further clinical tests directed towards the development of implantable corneal epithelium tissue.
